# Myasthenia Gravis and Pregnancy: Clinical Management and Maternal–Neonatal Outcomes

**DOI:** 10.3390/medicina62071238

**Published:** 2026-06-26

**Authors:** Zlatko Kirovakov, Angel Yordanov, Vasilena Dimitrova

**Affiliations:** 1 Faculty for Public Health and Healthcare, Prof. Assen Zlatarov University, 8010 Burgas, Bulgaria; kirovakov@yahoo.com; 2 Department of Gynecologic Oncology, Medical University Pleven, 5800 Pleven, Bulgaria

**Keywords:** myasthenia gravis, pregnancy, transient neonatal myasthenia gravis, thymectomy, pyridostigmine, immunotherapy, maternal outcomes, neonatal outcomes

## Abstract

*Background and Objectives*: To summarize current evidence regarding the pathophysiology, clinical management, and maternal and neonatal outcomes associated with pregnancy in women with myasthenia gravis (MG). *Materials and Methods*: This structured narrative review was based on literature searches conducted in major biomedical databases, including PubMed/MEDLINE, Scopus, Web of Science, and the Cochrane Library. Eligible publications included observational studies, cohort studies, case series, and clinically relevant case reports addressing MG management during pregnancy and postpartum. Due to heterogeneity in study design, patient populations, interventions, and reported outcomes, findings were synthesized narratively rather than quantitatively. *Results*: The available evidence suggests that pregnancy outcomes in women with MG are generally favorable when multidisciplinary monitoring and individualized treatment strategies are applied. Disease exacerbations may occur during pregnancy or the postpartum period, particularly in women with generalized MG or respiratory involvement. Observational evidence indicates that prior thymectomy may be associated with reduced rates of MG exacerbation and transient neonatal myasthenia gravis (TNMG), although available data remain limited. Careful selection of pharmacological therapy, prenatal monitoring, anesthetic management, and postpartum surveillance is essential to optimize maternal and neonatal outcomes. *Conclusions*: Myasthenia gravis is generally compatible with successful pregnancy outcomes; however, affected women may require closer monitoring because of the potential risk of disease exacerbation, myasthenic crisis, and neonatal complications. Management should be individualized and supported by multidisciplinary collaboration involving neurologists, obstetricians, anesthesiologists, and neonatologists.

## 1. Introduction

Myasthenia gravis (MG) is a chronic autoimmune disorder of the neuromuscular junction characterized by fluctuating skeletal muscle weakness resulting from impaired neuromuscular transmission. The disease is primarily mediated by immunoglobulin G (IgG) autoantibodies directed against postsynaptic acetylcholine receptors (AChRs), muscle-specific kinase (MuSK), or low-density lipoprotein receptor-related protein 4 (LRP4), leading to the disruption of normal neuromuscular signaling [1,2,3,4]. Clinically, MG most commonly affects the extraocular, bulbar, respiratory, and proximal limb muscles, resulting in symptoms such as ptosis, diplopia, dysphagia, dysarthria, and generalized muscle fatigue [5,6]. Although MG is considered a relatively rare disease, with an estimated prevalence of approximately 4 per 10,000 individuals, it occurs more frequently in women during the reproductive years, particularly in the second and third decades of life [7,8,9,10].

The coexistence of MG and pregnancy presents significant clinical challenges, as physiological, hormonal, and immunological changes during pregnancy may affect disease activity and maternal well-being. Disease exacerbations may occur during pregnancy or the postpartum period, whereas some women experience disease stability or temporary remission [11,12,13,14,15]. The management of MG during pregnancy requires a careful balance between maternal disease control and fetal safety, particularly when immunosuppressive therapies are required. Although medications such as pyridostigmine, corticosteroids, and azathioprine are generally considered acceptable in selected clinical settings, other immunosuppressive agents, including mycophenolate mofetil and methotrexate, are contraindicated because of teratogenic risk [16,17,18]. In severe or refractory cases, intravenous immunoglobulin (IVIG) and plasma exchange may provide effective therapeutic options [19].

Obstetric management of pregnant women with MG also requires individualized planning. Vaginal delivery is generally preferred when clinically feasible; however, maternal fatigue, bulbar weakness, or respiratory compromise may necessitate assisted vaginal delivery or cesarean section [20,21]. Anesthetic management is particularly important because several medications used during labor and delivery may impair neuromuscular transmission and worsen MG-related weakness [22,23,24]. Furthermore, the postpartum period is associated with an increased risk of disease exacerbation, necessitating close maternal monitoring and therapeutic adjustments when appropriate [25,26]. Neonatal complications, particularly transient neonatal myasthenia gravis (TNMG) caused by transplacental transfer of maternal antibodies, further emphasize the importance of coordinated multidisciplinary care involving neurologists, obstetricians, anesthesiologists, and neonatologists [27,28].

Despite growing clinical interest in pregnancy-associated MG, the available evidence remains limited and heterogeneous, consisting predominantly of retrospective studies, case series, and case reports. Variability in study design, treatment strategies, and reported maternal and neonatal outcomes complicates evidence synthesis and limits clinical decision-making. Although previous reviews have provided valuable clinical insights, important aspects including immunopathological mechanisms, treatment safety considerations, delivery management, and postpartum outcomes remain incompletely integrated in the existing literature. This structured narrative review aims to summarize current evidence regarding the pathophysiology, clinical management, maternal outcomes, and neonatal considerations associated with pregnancy in women with myasthenia gravis.

## 2. Materials and Methods

This structured narrative review aimed to summarize the current evidence regarding the pathophysiology, clinical management, maternal outcomes, and neonatal considerations associated with pregnancy in women with myasthenia gravis (MG). A structured literature search and narrative synthesis approach was used to identify and evaluate clinically relevant studies addressing MG during pregnancy and the postpartum period. The literature search was conducted using major biomedical databases, including PubMed/MEDLINE, Scopus, Web of Science, and the Cochrane Library. Additional relevant publications were identified through manual searches of reference lists. Searches included combinations of keywords and Medical Subject Headings (MeSH) related to “myasthenia gravis”, “pregnancy”, “maternal outcomes”, “neonatal myasthenia gravis”, “delivery”, “postpartum”, and “immunotherapy”. Eligible publications included observational studies, retrospective and prospective cohort studies, case series, and clinically relevant case reports addressing pregnancy-associated myasthenia gravis. Review articles and systematic reviews were consulted to provide contextual background and support interpretation of the findings but were not included in the primary evidence synthesis.

Conference abstracts, editorials, and publications lacking sufficient clinical relevance or outcome data were excluded. Due to the heterogeneity of available evidence, including differences in study design, patient populations, interventions, and outcome reporting, a formal quantitative synthesis and risk-of-bias assessment were not performed. Instead, findings were synthesized narratively with emphasis on clinically relevant patterns and management considerations. The review process was conducted to provide a clinically focused overview of the current literature rather than a formal systematic review or meta-analysis.

### 2.1. Eligibility Criteria and Information Sources

Studies were considered eligible if they addressed pregnancy or postpartum management in women diagnosed with myasthenia gravis (MG) and/or neonatal outcomes related to transient neonatal myasthenia gravis (TNMG). Relevant publications included studies evaluating pharmacological treatment, immunosuppressive therapy, intravenous immunoglobulin (IVIG), plasma exchange, anesthetic management, delivery planning, postpartum care, and multidisciplinary monitoring strategies. Maternal outcomes of interest included MG exacerbation, myasthenic crisis, mode of delivery, and anesthesia-related complications. Fetal and neonatal outcomes included preterm birth, intrauterine growth restriction, congenital abnormalities, perinatal complications, and TNMG. Additional clinically relevant considerations, including medication safety and breastfeeding, were also reviewed. Because of the limited availability of high-quality comparative studies in this field, observational studies, retrospective cohorts, case series, and clinically informative case reports were included. Conference abstracts, editorials, and publications lacking sufficient clinical relevance or outcome data were excluded. Major biomedical databases, including PubMed/MEDLINE, Scopus, Web of Science, and the Cochrane Library, were searched to identify relevant literature. Additional articles were identified through the manual screening of reference lists from eligible studies and relevant review articles.

### 2.2. Search Strategy and Screening Process

A structured literature search was conducted using major biomedical databases, including PubMed/MEDLINE, Scopus, Web of Science Core Collection, the Cochrane Library, and Google Scholar. Searches were performed using combinations of Medical Subject Headings (MeSH) and relevant keywords related to myasthenia gravis and pregnancy, including “myasthenia gravis”, “pregnancy”, “maternal outcomes”, “fetal outcomes”, “postpartum”, “delivery”, “neonatal myasthenia gravis”, and “immunotherapy”. Boolean operators (“AND” and “OR”) were used to combine search terms where appropriate.

Titles, abstracts, and when necessary, full texts, were screened for relevance according to the predefined eligibility criteria. Duplicate records were removed prior to screening. Additional relevant studies were identified through manual searches of reference lists from eligible articles and relevant review papers.

Studies were selected based on their clinical relevance to MG management during pregnancy, maternal and neonatal outcomes, delivery planning, anesthetic considerations, and postpartum care. Given the heterogeneity of the available literature and the narrative nature of the review, the findings were synthesized descriptively rather than quantitatively.

### 2.3. Data Extraction and Synthesis

Relevant information extracted from eligible studies included author, year of publication, study design, patient population, therapeutic interventions, maternal and neonatal outcomes, and key clinical findings. Because of substantial heterogeneity in study design, sample size, interventions, and reported outcomes, a formal quantitative synthesis and standardized risk-of-bias assessment were not performed. Instead, studies were evaluated narratively with consideration of their clinical relevance, methodological characteristics, and potential limitations. Findings were summarized descriptively and organized into thematic categories related to disease management, pregnancy outcomes, delivery considerations, postpartum care, and neonatal complications associated with myasthenia gravis. Review articles and systematic reviews were used exclusively as background references and were not considered part of the primary evidence synthesis to avoid duplication of evidence.

### 2.4. Methodological Approach

This review was designed as a structured narrative review rather than a systematic review or scoping review. Therefore, no formal PRISMA-based systematic review protocol, meta-analysis, or quantitative risk-of-bias assessment was performed. The literature search was conducted in a structured manner to identify clinically relevant evidence on myasthenia gravis during pregnancy and the postpartum period. Because the available literature is heterogeneous and includes cohort studies, retrospective analyses, case reports, and selected review articles, the findings were synthesized narratively with emphasis on clinical relevance, consistency of findings, and methodological limitations. The completed PRISMA 2020 Checklist is provided as Supplementary Material [29].

### 2.5. Assessment of Evidence

The strength of evidence was considered qualitatively according to study design, sample size, clinical relevance, and potential sources of bias. Population-based and cohort studies were interpreted as providing stronger observational evidence than single case reports or small case series. Case reports were used primarily to illustrate uncommon clinical scenarios and management considerations rather than to support generalized clinical recommendations. Review articles and systematic reviews were used for contextual background and were not considered primary evidence. Conclusions were therefore formulated cautiously and interpreted in light of the limited and heterogeneous evidence base.

## 3. Results

**Figure 1:**
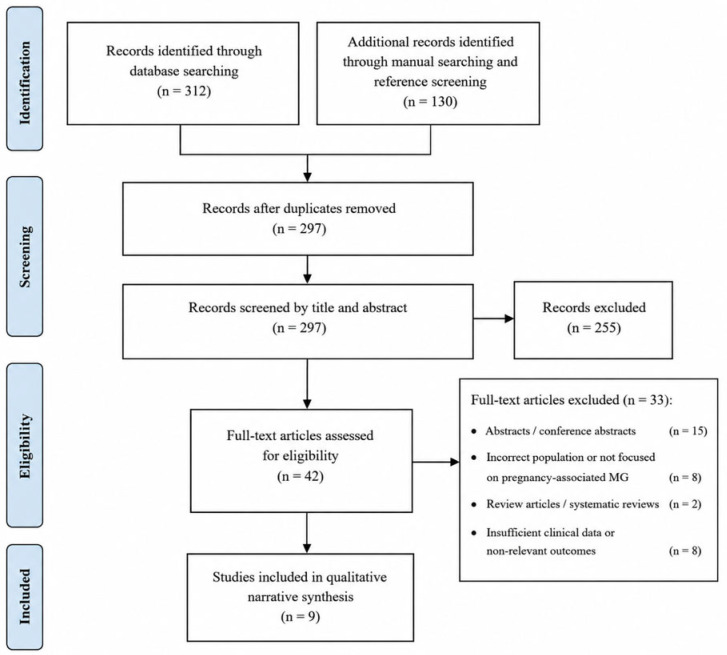
Flow diagram of the literature search, screening, and study selection process.

The literature search identified 312 records from electronic databases and an additional 130 records through manual searches, reference list screening, and other supplementary sources. After the removal of duplicate records and an initial screening for relevance, full-text articles were assessed for eligibility according to the predefined inclusion and exclusion criteria. A total of 9 primary studies were included in the final narrative synthesis. The included publications consisted of observational cohort studies, retrospective analyses, observational studies, and clinically informative case reports evaluating maternal disease course, pharmacological management, delivery considerations, and maternal and neonatal outcomes in women with myasthenia gravis (Figure 1 and Table 1).

## 4. Discussion

### 4.1. Clinical Characteristics and Disease Activity

Myasthenia gravis (MG) is a chronic autoimmune disorder characterized by fluctuating skeletal muscle weakness caused by impaired neuromuscular transmission. Clinical manifestations range from isolated ocular involvement to generalized disease affecting bulbar, respiratory, and proximal limb muscles. Ocular MG commonly presents with ptosis and diplopia and is generally associated with a milder clinical course, whereas generalized MG may result in significant functional impairment and increased risk of respiratory compromise [27,35,36]. Pregnancy may influence the clinical course of MG due to physiological, hormonal, and immunological changes occurring throughout gestation and the postpartum period. Available evidence suggests that disease exacerbations may occur during pregnancy, particularly during the first trimester and the early postpartum period, although some women experience stable disease or temporary remission [13,14,15,16]. Reported factors associated with MG exacerbation include respiratory muscle weakness, pregnancy-related hypoventilation, infections, medication adjustments, and physiological stress during labor and delivery [30,37].

The immunological profile of MG also varies according to antibody subtype. Acetylcholine receptor (AChR) antibodies are detected in approximately 80–90% of patients with generalized MG and in a lower proportion of patients with ocular disease [35]. Anti-muscle-specific kinase (MuSK) antibodies and antibodies directed against low-density lipoprotein receptor-related protein 4 (LRP4) have also been described in seronegative MG subtypes [38,39]. These immunological differences may contribute to variability in disease severity and treatment response. In addition, congenital myas-thenic syndromes should be considered in the differential diagnosis of early-onset or atypical neuromuscular junction disorders, although these conditions differ from autoimmune MG in their genetic background and clinical management [40,41,42].

### 4.2. Maternal Monitoring and Pharmacological Management

Management of MG during pregnancy requires individualized assessment and multidisciplinary collaboration involving neurologists, obstetricians, anesthesiologists, and neonatologists. The primary therapeutic objective is to maintain optimal maternal disease control while minimizing fetal risk associated with treatment exposure [11,12]. Pyridostigmine remains the mainstay of symptomatic treatment and is generally considered safe during pregnancy when clinically indicated [18]. Corticosteroids and azathioprine may also be used in selected patients, although treatment decisions should be individualized according to disease severity and maternal–fetal considerations [16,17,18,19]. In contrast, medications such as methotrexate and mycophenolate mofetil are contraindicated because of their known teratogenic effects [17,18]. In severe or refractory disease, intravenous immunoglobulin (IVIG) and plasma exchange may provide effective short-term disease stabilization [19]. Observational evidence suggests that prior thymectomy may be associated with lower rates of MG exacerbation and transient neonatal myasthenia gravis (TNMG), although available data remain limited and largely retrospective [43,44,45]. Similarly, the use of biologic therapies such as rituximab requires careful individualized evaluation because evidence regarding safety during pregnancy remains limited [31,46,47].

Women with active or generalized MG may require closer prenatal monitoring, particularly in the presence of bulbar or respiratory symptoms [48]. However, the optimal frequency of antenatal assessment has not been clearly established because current evidence is derived primarily from retrospective studies and case reports [32,49,50,51].

### 4.3. Preconception and Antenatal Counselling

Preconception counselling represents an important component of care for women with MG who are planning pregnancy. Counselling should include a discussion of disease stability, medication safety, potential pregnancy-related exacerbations, delivery planning, breastfeeding considerations, and neonatal risks, including TNMG [11,12,25]. Women should be informed that pregnancy outcomes are generally favorable when MG is adequately controlled and multidisciplinary care is available. Shared decision-making is essential, particularly regarding the continuation or modification of immunosuppressive therapy before conception and during pregnancy. Patients with significant bulbar or respiratory involvement may require additional counselling regarding potential maternal and fetal risks [52,53]. Psychological support and family involvement may also contribute positively to disease management during pregnancy and the postpartum period, particularly in women with severe disease or previous pregnancy complications.

### 4.4. Labor, Delivery, and Anesthetic Considerations

Delivery planning in women with MG should be individualized according to disease severity, respiratory status, and maternal functional capacity. Vaginal delivery is generally preferred when clinically feasible because uterine smooth muscle is not directly affected by MG [21]. However, prolonged labor, maternal fatigue, bulbar weakness, or respiratory compromise may necessitate assisted vaginal delivery or cesarean section [21,22,23,24]. Anesthetic management requires particular caution because several medications commonly used during labor and delivery may impair neuromuscular transmission. Regional anesthesia is generally preferred when appropriate, while medications including magnesium sulfate, neuromuscular blocking agents, aminoglycosides, and excessive sedative agents should be avoided or used cautiously because of the potential risk of worsening neuromuscular weakness [22,23,24].

**Figure 2:**
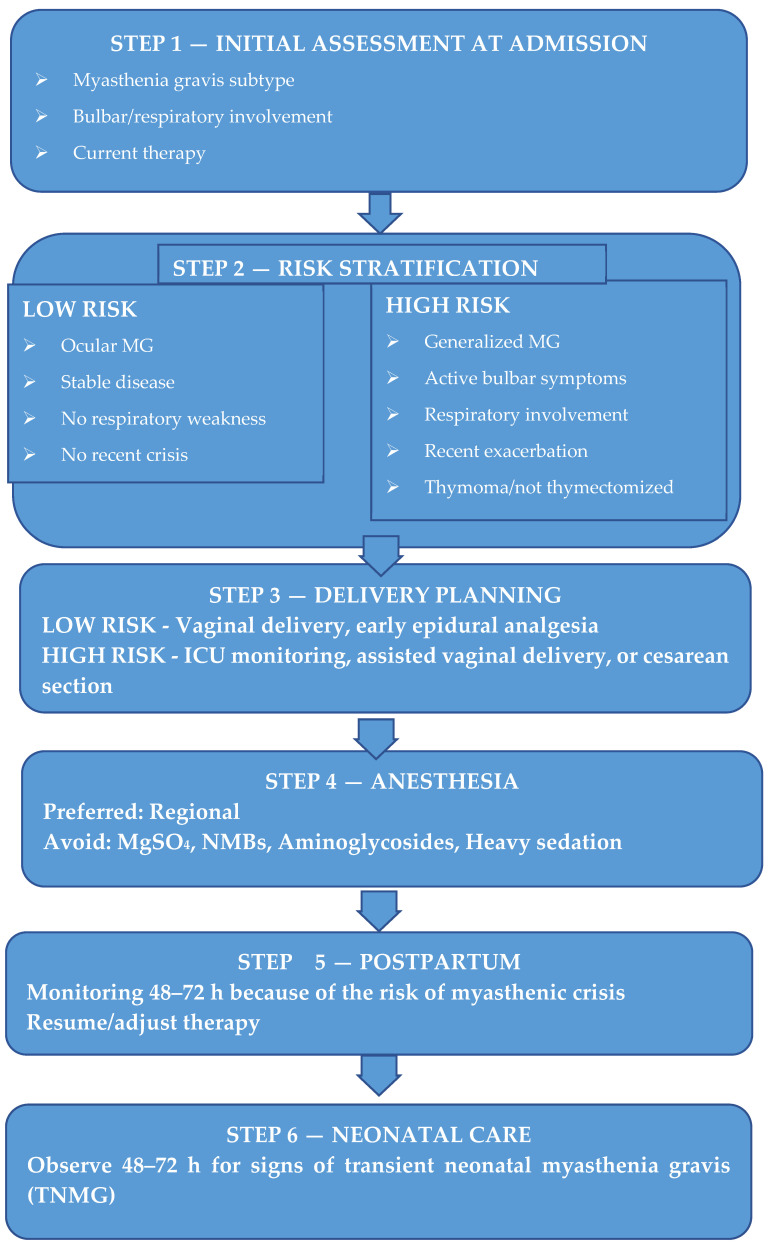
Risk-based algorithm for labor, delivery, and postpartum management in women with myasthenia gravis.

Postpartum monitoring is also important because disease exacerbations may occur during the first days after delivery [25,26]. Women with generalized MG or recent disease instability may require closer neurological observation and the adjustment of therapy during this period. Based on the evidence synthesized in this review, a structured clinical management algorithm is proposed to illustrate potential approaches to labor and postpartum care in women with MG (Figure 2).

The proposed framework represents an illustrative interpretation of the available literature rather than a validated guideline-based recommendation.

### 4.5. Neonatal Outcomes and Transient Neonatal Myasthenia Gravis

Transient neonatal myasthenia gravis (TNMG) is a temporary neuromuscular disorder caused by transplacental transfer of maternal IgG antibodies directed against components of the neuromuscular junction [27]. Affected neonates may present within the first days after birth with hypotonia, weak cry, poor feeding, respiratory distress, or generalized weakness.

Clinical manifestations are usually self-limiting because maternal antibodies gradually disappear from the neonatal circulation over time. Most cases resolve within several weeks with supportive management alone [27,28]. Neonatal observation during the first 48–72 h after delivery is generally recommended, particularly in symptomatic infants or in pregnancies complicated by severe maternal MG. Although antibodies directed against AChR and MuSK have been most commonly associated with TNMG, the relationship between other MG-associated antibodies and neonatal manifestations remains incompletely understood and requires further investigation [27,38,39].

The findings of the present review are consistent with conclusions from recent systematic reviews, which have emphasized the importance of multidisciplinary care, careful treatment adjustment during pregnancy, and close maternal and neonatal monitoring in women with myasthenia gravis [27].

### 4.6. Limitations

This review has several limitations. First, the narrative design and absence of formal risk-of-bias assessment limit the strength of the conclusions. Second, the available literature consists predominantly of retrospective studies, case series, and case reports, resulting in substantial heterogeneity across study populations, interventions, and reported outcomes. Publication bias may also be present, particularly because severe or unusual clinical presentations are more likely to be reported. In addition, the limited number of high-quality prospective studies restricts the ability to formulate definitive evidence-based recommendations.

## 5. Conclusions

Myasthenia gravis (MG) presents important clinical challenges during pregnancy because the disease primarily affects women of reproductive age and may influence maternal, fetal, and neonatal outcomes. Although most women with MG experience successful pregnancies, disease exacerbations, respiratory compromise, and postpartum deterioration may occur, particularly in patients with generalized disease or significant bulbar involvement. Management during pregnancy requires individualized therapeutic planning that balances maternal disease control with fetal safety. Pyridostigmine, corticosteroids, azathioprine, intravenous immunoglobulin (IVIG), and plasma exchange may be used in selected clinical settings, whereas teratogenic immunosuppressive agents such as methotrexate and mycophenolate mofetil should generally be avoided during pregnancy. Careful obstetric and neurological monitoring is particularly important during labor and the postpartum period because physiological stress and medication-related factors may precipitate disease worsening. Preconception counselling and multidisciplinary collaboration involving neurologists, obstetricians, anesthesiologists, and neonatologists remain central components of care. Women should be informed about potential maternal and neonatal risks, medication safety considerations, delivery planning, breastfeeding, and the possibility of transient neonatal myasthenia gravis (TNMG). Overall, the available evidence suggests that MG is generally compatible with favorable pregnancy outcomes when appropriate monitoring and individualized management strategies are applied. However, the current evidence base remains limited by the predominance of retrospective studies, case series, and case reports. Further prospective studies are needed to improve the understanding of optimal therapeutic strategies and maternal–neonatal outcomes in pregnancy-associated MG.

## Figures and Tables

**Table 1:** Overview of studies on myasthenia gravis and pregnancy included in this structured narrative review.

No.	Author/Year	Study Design	Population	Intervention/Management	Key Findings
1	Benlghazi et al. (2024) [14]	Case report	28-year-old pregnant woman with MG	Pyridostigmine, azathioprine, prednisone, thymectomy	Favorable postpartum recovery without disease decompensation; stable clinical follow-up over 1 year
2	Draxler et al. (2023) [30]	Retrospective cohort study	66 pregnant women with MG	Standard MG therapy	Generally favorable maternal and neonatal outcomes with low rates of TNMG and severe exacerbations
3	Hassan (2017) [31]	Case report	Pregnant woman with MG and preeclampsia	Cesarean delivery, spinal anesthesia, pyridostigmine, IVIG, antihypertensive therapy	Stable maternal neurological status and successful postpartum recovery
4	Neykova et al. (2022) [32]	Retrospective observational study	Five pregnant women with MG and COVID-19 infection	Baseline MG therapy with individualized adjustments	All patients recovered and delivered healthy neonates; pyridostigmine dose escalation required in selected cases
5	O’Connor et al. (2024) [8]	Nationwide population-based cohort study	443 pregnancies in women with MG	Obstetric and neurological monitoring	Increased rates of induced labor and cesarean section compared with controls; generally favorable neonatal outcomes
6	Ozcan et al. (2015) [9]	Case report	Pregnant woman with newly diagnosed MG and preeclampsia	Prednisolone, pyridostigmine, multidisciplinary monitoring	Maternal neurological improvement and uncomplicated neonatal recovery
7	Plancha et al. (2025) [15]	Case report	Pregnant woman with untreated MG	IVIG and pyridostigmine	Rapid symptom improvement and favorable neonatal outcome
8	Silvestri et al. (2024) [33]	Observational study	Pregnant patients with generalized MG	Ravulizumab or efgartigimod therapy	Ravulizumab associated with reduced concomitant medication use and less frequent dosing
9	Bahat et al. (2018) [34]	Case report	Pregnant woman with MG complicated by HELLP syndrome	Pyridostigmine, intensive care monitoring	Successful maternal stabilization and recovery following delivery

## Data Availability

The original contributions presented in this study are included in the article/Supplementary Material. Further inquiries can be directed to the corresponding authors.

## Supplementary Materials

The following supporting information can be downloaded at: https://www.mdpi.com/article/10.3390/medicina62071238/s1, PRISMA 2022 Checkllist.
